# Metformin Reverses Hashimoto’s Thyroiditis by Regulating Key Immune Events

**DOI:** 10.3389/fcell.2021.685522

**Published:** 2021-05-28

**Authors:** Xi Jia, Tianyu Zhai, Chunjie Qu, Jianjun Ye, Jing Zhao, Xuerong Liu, Jin-an Zhang, Qiaohui Qian

**Affiliations:** ^1^Department of Endocrinology, Shanghai University of Medicine and Health Sciences Affiliated Zhoupu Hospital, Shanghai, China; ^2^Shanghai Pudong New Area Center for Disease Control, Shanghai, China; ^3^Shanghai Kangqiao Community Health Service Center, Shanghai, China

**Keywords:** Hashimoto’s thyroiditis, metformin, Th17 cells, intestinal flora, immune

## Abstract

**Background:**

Hashimoto’s thyroiditis (HT) is a common autoimmune disease characterized by high levels of thyroid peroxidase antibody (TPOAb) and thyroid globulin antibody (TgAb) as well as infiltration of lymphocytes in thyroid. In recent years, metformin has been proven to be effective in a variety of autoimmune diseases, such as systemic lupus erythematosus, rheumatoid arthritis and multiple sclerosis.

**Methods:**

This study systematically explored the therapeutic effect of metformin on HT and its underlying mechanism by comprehensively utilizing methods including animal model, *in vitro* cell culture and differentiation, mRNA sequencing and 16S rRNA sequencing.

**Findings:**

We found that metformin indeed had a therapeutic effect on mice with HT mainly by reducing TgAb and lymphocyte infiltration in thyroid tissue. In addition, metformin also significantly suppressed the number and function of Th17 cells and M1 macrophages polarization in HT mice. Furthermore, metformin can inhibit the differentiation and function of Th17 *in vitro*. The results of mRNA sequencing of thyroid tissue illustrated that the therapeutic effect of metformin on HT was mainly achieved by regulating immune pathways. 16S RNA sequencing of the intestinal flora found that the intestinal flora of HT mice differs significantly from that of the normal mice and also were altered by metformin treatment.

**Interpretation:**

These experiments provided a preliminary theoretical basis for the clinical application of metformin in the treatment of HT.

## Introduction

Hashimoto’s thyroiditis (HT) is an organ-specific autoimmune disease susceptible to women with genetic susceptibility, mainly causing hypothyroidism (Viard and Gilquin, 2017). HT is characterized by lymphocytic infiltration of the thyroid tissue accompanied by the production of specific antibodies, among which, thyroid peroxidase antibodies (TPOAb) and thyroglobulin antibodies (TgAb) are the most important pathogenic antibodies (Antonelli et al., 2015; Wang et al., 2018). Autoimmune diseases are often multifactorial and have an unclear pathogenesis. It is believed that genetic factors, autoimmunity and environmental factors are involved in the pathogenesis of HT, in which the destruction of autoimmune tolerance of thyroid caused plays a key role (Qin et al., 2012; Taylor et al., 2018; Li et al., 2019).

Metformin (dimethyl biguanide), a widely used biguanide oral hypoglycemic agent, was first discovered in the herbal *Galega officinalis*. Since its inception more than 50 years ago, metformin has marked a global milestone in the treatment of type 2 diabetes mellitus (T2DM) (Marcucci et al., 2020). Recent evidence suggests that metformin has a novel pleiotropic effect. In addition to playing an important role in diabetes, it also exhibits cardiopulmonary and kidney protection as well as anti-proliferative, anti-fibrotic and anti-oxidative effects (Fujita and Inagaki, 2017; Nafisa et al., 2018; Vancura et al., 2018; Soukas et al., 2019). Moreover, emerging ground-breaking studies support the new hypothesis that metformin possesses immunomodulatory features both *in vivo* and *in vitro* (Schuiveling et al., 2018; Ursini et al., 2018). Studies have shown that metformin can interfere with important immunopathological mechanisms associated with systemic autoimmune diseases in a variety of ways, including T helper 17/T regulatory cell balance, macrophage polarization, germinal center formation, autoantibody production, cytokine secretion, etc. (Schuiveling et al., 2018; Ursini et al., 2018). The immunomodulatory properties of metformin have also attracted many scientists to explore its role in a variety of autoimmune diseases. Currently, metformin has been shown to have therapeutic effects on a variety of autoimmune diseases in animal models, including systemic lupus erythematosus (SLE) (Wang et al., 2015), rheumatoid arthritis (RA) (Lu et al., 2019; Rajaei et al., 2019), inflammatory bowel disease (IBD) (Lee et al., 2015) and multiple sclerosis (MS) (Sun et al., 2016). Among them, metformin therapy for SLE has been proven effective in clinical trials (Sun et al., 2020). The mechanism by which metformin treats these autoimmune diseases is multifaceted, involving the reconstitution of immune system homeostasis, regulation of 5’-AMP-activated protein kinase (AMPK)- mammalian target of rapamycin (mTOR) signaling pathways and improving gut microbe metabolism (Ursini et al., 2018).

We have previously performed a meta-analysis of 75 patients with HT and 100 patients with subclinical thyroiditis (SH) and found that metformin effectively reduced the levels of TPOAb and TgAb in both HT and SH patients, and significantly inhibited thyroid stimulating hormone (TSH) level (Jia et al., 2020). Based on the above results, we hypothesized that metformin may have a potential therapeutic effect on HT via pleiotropic mechanisms, which need to be verified from multiple perspectives.

## Materials and Methods

### Preparation of Animal HT Model

**FIGURE 1:**
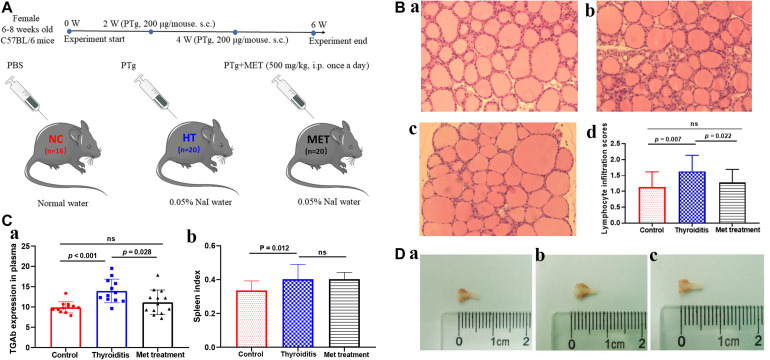
**(A)** Animal model experiment process. **(B)** H&E staining of thyroid tissue, **(a)** control, **(b)** Thyroiditis, **(c)** Metformin treated, **(d)** lymphocyte infiltration scores (ns, not significant). **(C)** TGAb expression in plasma and spleen index comparison. **(D)** Mouse thyroid tissue, **(a)** control, **(b)** Thyroiditis, **(c)** Metformin treated.

In this experiment, a mouse HT model was constructed by high-iodine water feeding and thyroglobulin immuno-injection. In brief, 56 female 6–8 weeks old C57BL/6 mice from Shanghai Model Organisms Center were kept at the SPF level and free to eat and drink. They were randomly divided into control group (*n* = 16), disease model group (*n* = 20) and metformin treatment group (*n* = 20). Mice in the disease and metformin groups were fed with 0.05% NaI water and accepted multiple injections of porcine thyroglobulin (200 μg/mouse) and complete Freund’s adjuvant on the back, abdomen and neck during the first week of modeling and injection of porcine thyroglobulin (200 μg/mouse) and incomplete Freund’s adjuvant 2 weeks later. Simultaneously, mice in the metformin treatment group were intraperitoneally injected with 500 mg/kg (dissolved in PBS) metformin once a day and mice in the HT model group was intraperitoneally injected with the same amount of PBS from the first immunization to the end of the modeling. All mice were sacrificed 4 weeks after the second immunization. All animal experiment procedures were approved by the Animal Ethics Committee of Zhoupu hospital. The flow of animal experiments is presented in Figure 1A.

### Pathologic Assessment of Autoimmune Thyroiditis

The mouse thyroid tissue was taken under a microscope, quickly fixed in 4% paraformaldehyde and embedded in paraffin. The thyroid tissue paraffin blocks were cut into 4 μm sections, stained with hematoxylin and eosin (H&E) and observed under a 200× light microscope. Lymphocyte infiltration score was determined by two members of the research group according to the percentage of lymphocyte infiltration area as the followings: 0, normal; 1+, 1–10%; 2+, 10–30%; 3+, 30–50%; 4+, and >50% (Li et al., 2017). Three fields of view of each sample were observed and the average was taken as the final score.

### Enzyme-Linked Immunosorbent Assay (ELISA) of Plasma IL-17

A total of 1–1.5 ml of whole blood was collected using retrobulbar bleeding method under anesthesia in an ethylenediaminetetraacetic acid anticoagulant EP tube and centrifuged at 2,000 rpm for 5 min at 4°C to obtain plasma. The expression levels of TgAb (Cat # CSB-E09543m, Aino-American Biotechnology Co., Ltd.) and IL-17 (Cat # M1700, R&D) in mouse plasma were determined using ELISA according to the manufacturer’s recommended protocols.

### Flow Cytometric Analysis of Mouse Spleen Cells

The entire spleen of each mouse was obtained, weighed, and placed on a 200-mesh sterile cell sieve. After slowly mixed with 20 ml of RPMI 1640 cell culture medium (containing 10% inactivated fetal bovine serum), each spleen was ground and meshed into very fine parts. The cell suspension was centrifuged. After removal of the supernatant, cell pellets were resuspended in red blood cell lysis buffer to obtain single splenic cell suspension. The spleen lymphocyte mononuclear cells were counted after trypan blue staining and adjusted to 5 × 10^6^/test. The cells were cultured for 5 h in 6-well plates with each well containing 2 ml of RPMI 1640 cell culture medium supplemented with 10% inactivated fetal bovine serum and 1% penicillin + streptomycin as well as 2 μl of cytokine stimulating agent. To determine the percentage of Th1 cells, Th2 cells and Th17 lymphocytes, the resulting spleen cells were labeled with CD3 antibodies (Cat # 145-2C11, BD Pharmingen, CA) and CD8 antibodies (Cat # 551162, BD Pharmingen, CA), membrane ruptured, and used to measure IL-4, IL-17 and IFN-γ.

We also detected macrophage polarization by labeling M1 macrophage as CD45^+^CD11b^+^Ly6G^–^F4/F80^+^CD206^–^ and M2 macrophage as CD45^+^CD11b^+^ Ly6G^–^F4/F80^+^CD206^+^ (Misharin et al., 2013). In brief, cells samples were labeled with antibodies CD45-APC-Cy7 (Cat # 565853, BD Pharmingen, CA), CD11b-PE-Cy7 (Cat # 101216, BioLegend, CA), Ly6G-BV421 (Cat # 562737, BD Pharmingen, CA) and F4/80-Alexa Fluor (Cat # 565853, BD Pharmingen, CA) for 20 min at 4°C in the dark. After that, cells were mixed first with lysis buffer then with antibody CD206-PE (Cat # 141706, BioLegend, CA) at 4°C for 25 min in the dark for intracellular staining. The prepared cell suspension was filtered through a sterile 300 mesh filter to remove cell pellets and then subjected to flow cytometric analysis (Beckman CytoFlex).

### Thyroid Immunohistochemistry and Immunofluorescence Analysis

Mouse thyroid tissues were prepared as 4 μm paraffin sections. After antigen retrieval, each section was treated with 100 μL of 3% H_2_O_2_ for 10 min at room temperature and blocked with 100 μL of 2.5% goat serum blocking solution for 30 min at room temperature. For immunohistochemistry analysis, after removal of the blocking solution, each section was incubated first for 1 h with 100 μL of diluted primary antibodies (IL-17A antibody, 1:500, Cat # NBP1-76337, Novus; CD68/SR-D1 Antibody, 1:150, Cat # NB100-683, Novus) then with 100 μL of secondary antibody for 30 min at room temperature. The positive cells were revealed by incubating with 100 μL of freshly prepared 3,3’-diaminobenzidine (DAB) solution. For immunofluorescence analysis, after removal of the blocking solution, each section was incubated first with 100 μL of diluted primary antibody (IL-17A antibody, 1:400, Cat # NBP1-76337, Novus) overnight at 4°C then with 100 μL of secondary antibody for 2 h at room temperature in the dark. Cells were counterstained with 100 μL 4′,6-diamidino-2-phenylindole (DAPI) for 10 min in the dark.

### Mouse Thyroid Tissue mRNA Sequencing

Five mice from disease model group, six from metformin treatment group and three from normal control mice were selected for thyroid tissue mRNA sequencing analysis. In brief, mouse thyroid tissues were taken under the microscope after carefully removing tracheal and other connective tissues around the thyroid gland, rapidly ground in a homogenizer containing ceramic beads and 1 ml of Trizol to a clear, flocculated form, and frozen at −80°C. Total RNA was extracted using the conventional Trizol-chloroform method and their concentration, 28S/18S ratio, and RNA integrity number (RIN) were measured using Agilent RNA 6000 Nano Kit on an Agilent 2100 Bioanalyzer. These RNA were subjected to 8G flux mRNA-seq on the BGI SEQ-500 second generation sequencing platform following the standard procedures. The raw data were cleaned to filter out reads with low quality, joint contamination and unknown base N. The clean reads were then aligned to the reference genome. If new transcripts were identified, those with protein coding potential were added to the reference gene sequence to form a complete reference database, which was used to calculate gene expression level or fragments per kilobase of transcript per million mapped reads (FPKM). Subsequently, differences in expression level between groups were analyzed using the DEGseq method, and genes with fold change ≥ 2 and adjusted *P*-value ≤ 0.001 were considered as differentially expressed genes and subjected to gene ontology (GO) enrichment analysis and Kyoto encyclopedia of genes and genomes (KEGG) pathway analysis using Phyper function of R software and cluster analysis using the Pheatmap function. This part of the experiment was carried out according to the common procedure of mRNA sequencing, and strict quality control was carried out (Kukurba and Montgomery, 2015).

### Induced Differentiation, Enrichment, and Metformin Stimulation of Th17 Cells

Spleen monocytes were obtained as described above. CD4^+^ T cells were separated from these cells using CD4^+^ T cell microbeads. CD4^+^CD44^*low*^CD62L^*high*^ naïve T cells were sorted out by flow cytometry and differentiated into Th17 cells in the presence of anti-CD3 and anti-CD28 coated beads as well as IL-6 (20 ng/ml; Cat # P4364, Novus), TGF-β1 (3 ng/ml; Cat # 763104, BioLegend), anti-IFNγ antibody (10 μg/ml; Cat # 517906, BioLegend), anti-IL-4 antibody (10 μg/ml; Cat # 504136, BioLegend), IL-23 (25 ng/ml; Cat # 589004, BioLegend) and IL-1β (20 ng/ml; Cat # 575104, BioLegend) for 48 h (Hasan et al., 2017). The differentiated Th17 cells were then stimulated with 20 ng/ml PMA and 1 μg/ml ionomycin for 30 min. Living IL-17 cells were captured and enriched using microbeads and labeled with phycoerythrin using a mouse IL-17 secretion assay kit (Cat # 130-094-213, Miltenyi, CA) according to manufacturer’s instructions and previous studies (Hasan et al., 2017; Venken et al., 2019). The phycoerythrin-labeled IL-17-secreting cells were sorted by flow cytometry.

In the above Th17 cells inducing process, metformin (0, 0.5, and 5 mmol/L; Cat # 317240, Sigma-Aldrich, Germany) was added at the beginning and its effects on Th17 cells differentiation in each group was analyzed by flow cytometry. The setting of the dose gradient of metformin referred to a previous study (Limagne et al., 2017). In brief, Th17 cells obtained without metformin intervention were collected by flow cytometry sorting and cultured in a 48-well plate. IL-17 secreting cells obtained from same mouse were equally divided into two groups and cultured for 1 day without intervention. After reaching stable status, cells were treated with metformin (0 and 5 mmol/L). After 8 h of treatment, cells were collected and used for flow cytometry and mRNA transcriptome sequencing (method described above).

### Mouse Intestinal Flora Detection by 16S Sequencing

Upon establishment of Hashimoto’s thyroiditis animal model and completion of metformin intervention (within 30 min before sacrifice), mice were grabbed and their abdomens were gently massaged to stimulate defecation. A total of 4 fresh feces per mouse were collected in a sterile cryopreservation tube and snap frozen in liquid nitrogen for future use.

Fecal DNA were extracted. Their quality and quantity were examined using Qubit Fluorometer instrument and DNA Broad Range kit and on a 1% agarose gel. Qualified samples were used for 16S rRNA sequencing library construction. In brief, the V3–V4 hypervariable region of 16s rRNA gene was amplified using Klenow DNA polymerase. After end repair and dA-tailing using T4 DNA polymerase and T4 polynucleotide kinase, the amplicon was ligated to T-tailed adaptors. The obtained libraries were purified and subjected to quality control. The qualified libraries were then analyzed on the Illumina platform for paired-end sequencing. Raw data were filtered to obtain high-quality clean data for bioinformatic analysis. The specific principles and procedures of this method were described by previous studies (Sanschagrin and Yergeau, 2014; Johnson et al., 2019).

### Statistical Analysis

Continuous variables were calculated as mean ± standard error (SE) and analyzed using independent sample *t*-test to identify significant differences between groups. Non-normal distribution data were analyzed using Mann-Whitney *U*-test. Statistical analysis of data was performed using GraphPad Prism (8.0, GraphPad Software, United States) and STATA (release 15, StataCorp, United States) with *P*-values < 0.05 being considered to have significant statistical differences. All flow cytometry experimental data were analyzed using CytExpert (2.0, Backman, United States) and mRNA-sequencing results were analyzed using R software (version 3.5.1).

## Results

### Metformin Reduces the Severity of Autoimmune Thyroiditis in Mice

In this study, HT model mice and metformin treated mice were evaluated for TgAb titer, thyroid lymphocyte infiltration degree and spleen index. H&E staining showed obvious lymphocyte infiltration and follicular morphological change in thyroid tissue of HT mice. In addition, the lymphocyte infiltration score of HT mice was higher than that of normal mice (*P* = 0.007) and metformin-treated HT mice (*P* = 0.022; Figure 1B). ELISA results showed that TgAb titer was significantly higher in HT mice than in control mice (*P* < 0.001), and metformin treatment significantly reduced TgAb titer in HT mice (*P* = 0.028; Figure 1Ca). Moreover, spleen index was significantly higher in HT mice than in normal mice (*P* = 0.012). However, metformin treatment did not significantly reduce spleen index of HT mice (*P* > 0.05; Figure 1Cb). Furthermore, thyroid tissue volume of HT mice was larger than that of the normal mice and reduced after metformin treatment (Figure 1D).

### Metformin Reduces the Proportion of Th17 Cells Differentiated From Naïve T Cells and in Mice With Autoimmune Thyroiditis

**FIGURE 2:**
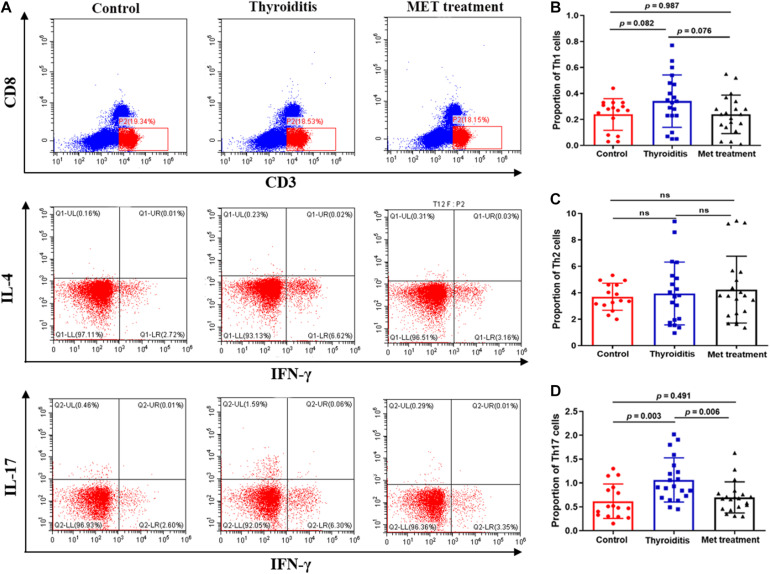
Flow cytometry and statistics of mouse spleen T cells; **(A)** Flow cytometry diagram, **(B)** Proportion of Th1 cells, **(C)** Proportion of Th2 cells, **(D)** Proportion of Th17 cells.

**FIGURE 3:**
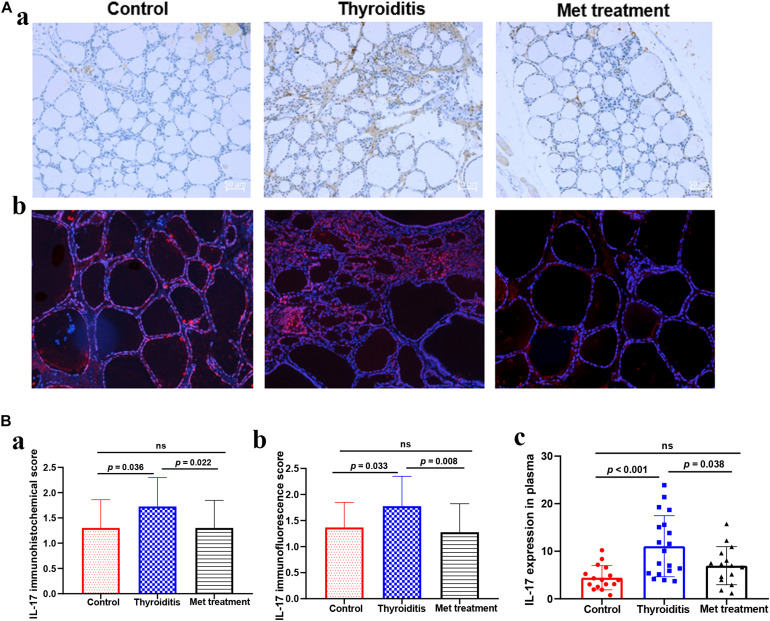
**(A)** IL-17 Immunohistochemistry and immunofluorescence of thyroid tissue, **(a)** IL-17 immunohistochemistry, **(b)** IL-17 immunofluorescence. **(B)** The expression level of IL-17 in thyroid tissue and plasma, **(a)** the immunohistochemical score of IL-17 in thyroid tissue, **(b)** the immunofluorescence score of IL-17 in thyroid tissue, **(c)** the expression level of IL-17 in plasma detected by ELISA.

We used flow cytometry to analyze Th1 cells, Th 2 cells and Th 17 cells in mouse spleen (Figure 2A). Flow cytometry analysis found that the changes in Th1 cells and Th2 cells were not significant in statistics (Figures 2B,C). However, the proportion of Th17 cells was significantly elevated in autoimmune thyroiditis mice (*P* = 0.003), but this elevation was decreased significantly after metformin treatment (*P* = 0.006) (Figure 2D). Moreover, IL-17 level was elevated in thyroid tissue of HT mice (*P*_*I*__*mmunohis*__*to*__*chemistry*_ = 0.036, *P*_*I*__*mmunofluorescence*_ = 0.033) and this elevation was reversed after metformin treatment (*P*_*Immunohis*__*to*__*chemistry*_ = 0.022, *P*_*Immunofluorescence*_ = 0.008; Figures 3A,Ba,Bb). Similarly, plasma IL-17 expression level was also significantly higher in HT mice than normal control mice (*P* < 0.001), and this elevation was significantly attenuated after metformin treatment (*P* = 0.038; Figure 3Bc).

**FIGURE 4:**
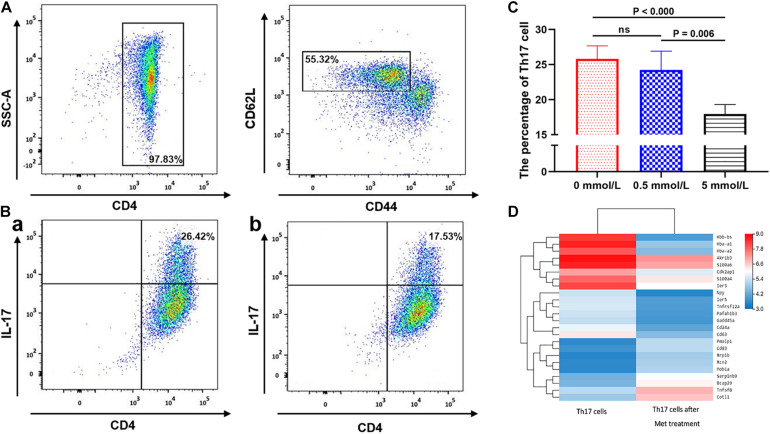
The effect of metformin on the differentiation of Naive T cells into Th17 cells. **(A)** Flow cytometry diagram of Naïve T cells. **(B)** Flow cytometry diagram of Th17 cells, **(a)** 0 mmol/L metformin, **(b)** 5 mmol/L metformin. **(C)** Proportion of Th17 cell differentiation under different metformin concentrations. **(D)** Differences in the mRNA transcriptome of Th17 cells before and after metformin treatment. The percentages shown in **(A,B)** were the mean value of the corresponding groups.

We further analyzed the inhibitory effect of metformin on Th17 cell differentiation. Flow cytometric analysis showed that the purity of the CD4^+^ T cells isolated using immunomagnetic beads was >95%, and the labeled CD44^*low*^CD62L^*high*^ naïve T cells accounted for 55.32 ± 0.23% (Figure 4A). Flow cytometric sorting of Th17 cells after using the above-mentioned Th17 cell induction protocol found that Th17 cells accounted for 26.42 ± 0.12% (mean ± SD%) in the absence of metformin, 26.28 ± 0.12% in the presence of 0.5 mmol/L metformin and only 17.53 ± 0.21% in the presence of 5 mmol/L metformin (*P* = 0.006; Figures 4B,C).

mRNA sequencing of Th17 cells in each group revealed that the transcriptome of Th17 cells changed significantly after metformin intervention. 14,132 genes were shared in Th17 cells with and without metformin intervention, while 1,620 genes belonged only to Th17 cells without metformin intervention, and 1,397 genes were newly detected in Th17 cells after metformin intervention Further KEGG pathway analysis and GO enrichment analysis of the differentially expressed genes found that most genes were enriched in the metabolic pathway, followed by the pentose and glucuronate conversion pathway, cytokine-cytokine receptor interaction and drug metabolism. After further screening of differentially expressed genes based on “expression level > 10 and differential expression log2 > 1.5,” a total of 24 differentially expressed genes were identified including CD63, CD83, and CD24a, etc., which were showed by heatmap (Figure 4D).

### Metformin Inhibits Macrophage Differentiation Into M1 Subset

**FIGURE 5:**
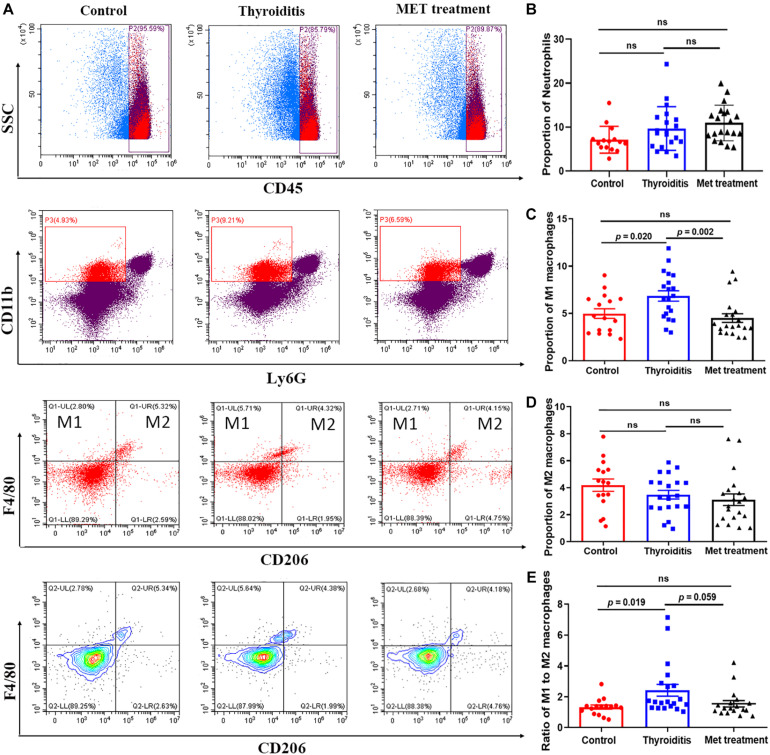
Flow cytometry and statistics of mouse spleen neutrophils and macrophages; **(A)** Flow cytometry diagram, **(B)** Proportion of neutrophils, **(C)** Proportion of M1 macrophages, **(D)** Proportion of M2 macrophages, **(E)** Ratio of M1–M2 macrophages.

Flow cytometric analysis also found significantly higher proportion of M1 macrophages and ratio of M1 and M2 cells in mice with autoimmune thyroiditis than in normal mice (*P* = 0.020 and *P* = 0.019, respectively; Figures 5A,C,E). After metformin treatment, the proportion of M1 macrophages decreased significantly (*P* = 0.002) and the ratio of M1 and M2 cells also decreased slightly (*P* = 0.059) (Figures 5A,C,E). However, the proportion of M2 macrophages was relatively stable, showing no significant difference among the three groups (Figure 5D). Immunohistochemistrical analysis of thyroid tissue labeled with CD68 showed no significant difference among the three groups of mice (data not shown). Furthermore, no significant differences were found in the percentage of neutrophils and CD45^+^ cells among the three groups (Figure 5B).

### Metformin Regulates Local Immunity of Thyroid Tissue

The transcriptome of mouse thyroid tissue changed significantly among the normal group, HT group and metformin treatment group, as shown in the volcano map of the differentially expressed genes (Supplementary Figure 1A). To further understand the function of these differentially expressed genes, GO enrichment and KEGG pathway enrichment analyses were performed (Supplementary Figures 1B,C). GO enrichment analysis showed that differentially expressed genes between HT mice and metformin treated mice were concentrated in immune system including immune response, innate immune response, T cell activation and differentiation as well as white blood cell activation and regulation. KEGG pathway enrichment analysis showed that these differentially expressed genes were mainly concentrated in immune system, specifically in Th17 cell differentiation, Th1 and Th2 cell differentiation, T cell receptor signaling pathways and other immune-related pathways. In addition, KEGG analysis also revealed that these differentially expressed genes were associated with many other autoimmune diseases, including type 1 diabetes, inflammatory bowel disease and rheumatoid arthritis (Supplementary Figure 1D).

### Metformin Changes the Intestinal Flora of Mice With Hashimoto’s Thyroiditis

**FIGURE 6:**
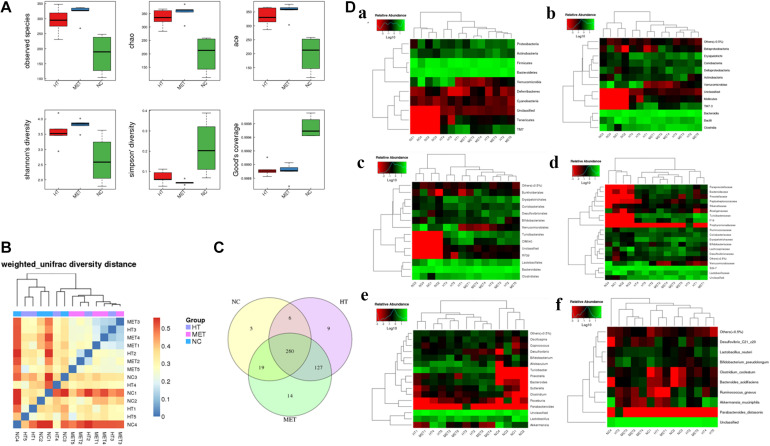
Differences in intestinal flora detected by 16s-RNA sequencing. **(A)** Differences in observed species, chao index, ace index, Shannon’s diversity, Simpson’ diversity and Good’s coverage between groups. **(B)** Weighted unifrac diversity distance. **(C)** Venn diagram of flora between groups. **(D)** Species profiling at different levels, **(a)** phylum classification level, **(b)** class classification level, **(c)** order classification level, **(d)** family classification level, **(e)** genus classification level, and **(f)** species classification level.

Alpha diversity and Beta diversity analyses found that the species index, chao index, ace index and Shannon’s diversity of the intestinal flora of HT mice and metformin treated mice were significantly higher than those of normal mice, while the Simpson index and Good’s coverage were significantly lower in the normal mice (Figure 6A). These results indicated that the species of intestinal flora of HT mice and metformin treated HT group were significantly more abundant. Weighted unifrac diversity distance and Venn diagram of flora between groups showed in Figures 6B,C. In addition, the intestinal flora of mice in each group was classified into phylum, class, order, family, genus and species based on operational taxonomic units (OUT) (Figure 6D). Compared with HT mice, the abundance of Coriobacteriia, Coriobacteriaceae, and Coriobacteriales in the intestinal flora of HT mice after metformin treatment decreased significantly, while that of Bacteroides and Butyricicoccus_pullicaecorum increased. Different intestinal flora of HT mice and metformin treated HT mice at different classification levels were summarized in Supplementary Table 1.

## Discussion

Metformin has been used to treat a variety of autoimmune diseases in animals and humans, such as SLE, RA, IBD, and MS (Ursini et al., 2018). However, its role in treatment of autoimmune thyroiditis has not been well investigated. This study systematically explored the therapeutic effect of metformin on HT and its underlying mechanism by comprehensively utilizing methods such as animal model, *in vitro* cell culture and differentiation, mRNA sequencing and 16S rRNA gene sequencing. First, an HT mouse animal model was established and treated with metformin. Second, the therapeutic effect of metformin was evaluated in terms of changes in TgAb titer, lymphocyte infiltration degree in thyroid tissue, and regulation of HT immune disorders. Third, mRNA sequencing was used to identify differentially expressed genes in thyroid tissues of normal mice, HT mice and metformin treated HT mice. Fourth, differences in intestinal flora between HT mice and normal mice and whether metformin has an effect on the intestinal flora of HT mice were explored using 16S rRNA sequencing. Our results for the first time proved that metformin can significantly decrease TgAb titer and reduce lymphocyte infiltration in thyroid tissue of HT mice, which provides direct evidence for the therapeutic effect of metformin on HT. Notably, TgAb is not the most accurate indicator of HT, because the level of antibodies may change during the disease (Ralli et al., 2020). However, due to strict control of interference factors in our experiment, the HT group and the metformin treatment group were considered in similar stages of disease course. Therefore, it can still reflect the therapeutic effect of metformin on HT.

The therapeutic effect of metformin on Hashimoto’s thyroiditis was mainly achieved by regulating immune disorders. In the present study, metformin shown a remarkable inhibitory effect on the activity and differentiation of Th17 cells, evidenced by the relatively lower levels of IL-17 in mouse thyroid tissue and plasma after metformin treatment, and lower proportion of Th 17 cells in the differentiation process of naïve T cells treated with metformin. Further mRNA sequencing found that metformin treatment significantly affected the metabolic pathway of Th17 cells and aldose reduction pathway. The inhibitory effect of metformin on Th17 cells is reported to be related to enhanced AMPK expression and suppressed mTOR-STAT3 signaling (Lee et al., 2017), but in our results, these genes did not differ significantly. On the other hand, hyperpolarization of M1 cells, a classically pro-inflammatory phenotype, were found in HT mice and significantly inhibited by metformin intervention. Metformin has been previously demonstrated to have anti-inflammatory properties by regulating the M1/M2 macrophage polarization in AMP-dependent and independent manners (Ursini et al., 2018). Interestingly, significant inhibitory effect of metformin on M1 macrophages was only observed in spleen cells but not in thyroid, since the expression difference of CD68, a major macrophage marker, was not significant in thyroid tissue. In order to further explore the local immunity changes in thyroid tissue, mRNA sequencing of mouse thyroid tissue was conducted and the results proved that the therapeutic effect of metformin on HT was mainly achieved by regulating immune pathways. However, although a variety of research methods, such as immunohistochemistry, immunofluorescence and mRNA sequencing of thyroid tissue, were used to study local immune changes in the thyroid, how the immune cells infiltrating the thyroid tissue were inhibited by metformin has still not been fully revealed. Therefore, more researches are needed to further study the effect of metformin on the local infiltrating macrophages and lymphocytes of the thyroid.

Disturbance of intestinal flora may play a key role in the development of Hashimoto’s thyroiditis. Previous studies have revealed many interrelationships in embryology, phylogeny and function between the thyroid and the gastrointestinal tract through the adulthood (Miller et al., 1978). The molecular mimicry mechanisms also provide a reasonable explanation for the role of intestinal flora in triggering autoimmune diseases (Benvenga and Guarneri, 2016; Sousa Mde et al., 2016; Figura et al., 2019). Changes in the composition of gut microbiota have been confirmed to be related to the development of several autoimmune diseases, including RA (Yeoh et al., 2013), SLE (Lopez et al., 2016), MS (Shamriz et al., 2016) and Behcet’s disease (Consolandi et al., 2015). In this experiment, we also studied the changes in the intestinal flora of HT mice and the effect of metformin on the intestinal flora of HT mice. The results showed significant differences in intestinal flora at different classification levels between HT mice and normal control mice, and metformin treatment also changed the intestinal flora of the HT mice such as Coriobacteriia, Coriobacteriaceae, Bacteroides, Coriobacteriales and Butyricicoccus_pullicaecorum. After metformin treatment, Bacteroides level in intestine of HT mice doubled, consistent with a previous study showing that metformin can significantly increase the level of Bacteroides (Zheng et al., 2020). In addition, our study found for the first time that metformin can reduce the levels of Coriobacteriia, Coriobacteriaceae and Coriobacteriales and increase the level of Butyricicoccus_pullicaecorum in the intestine of HT mice. However, the previously reported metformin-related bacteria, Akkermansia_muciniphila (de la Cuesta-Zuluaga et al., 2017), was not significantly different in HT mice and metformin treated mice.

In summary, our study has verified that metformin has a therapeutic effect on HT from multiple perspectives such as animal model, *in vitro* cell culture, mRNA sequencing and 16S rRNA sequencing. However, our study still has some shortcomings and needs to be further improved. The major limitation of the present research comes from the usage of only one animal model, while the large sample size of each group may partially compensate for this shortcoming. Moreover, our results could be more robust if clinical data were available to support the therapeutic effect of metformin on HT.

## Data Availability Statement

The datasets presented in this study can be found in online repositories. The names of the repository/repositories and accession number(s) can be found below: NCBI SRA (BioProject ID: PRJNA723153).

## Ethics Statement

The studies involving human participants were reviewed and approved by Ethical Committees of Zhoupu Hospital. The patients/participants provided their written informed consent to participate in this study. The animal study was reviewed and approved by Ethical Committees of Zhoupu Hospital.

## Author Contributions

XJ and TZ: design, operation and data analysis of the experiment. XL, JZ, and CQ: breeding and management of the experimental animals. J-AZ and QQ: overall direction of the research. All authors contributed to the article and approved the submitted version.

## Conflict of Interest

The authors declare that the research was conducted in the absence of any commercial or financial relationships that could be construed as a potential conflict of interest.

## Supplementary Material

Supplementary Figure 1 mRNA sequencing of mice thyroid tissue. **(A)** Thyroid tissue differential gene volcano map. **(a)** different genes of mice in NC group vs. HT group, **(b)** different genes of mice in HT group vs. Met treatment group; **(B)** GO enrichment bubble chart of differential genes in thyroid tissue. **(a)** different genes of mice in NC group vs. HT group, **(b)** different genes of mice in HT group vs. Met treatment group; **(C)** KEGG pathway classification of differential genes in thyroid tissue. **(a)** different genes of mice in NC group vs. HT group, **(b)** different genes of mice in HT group vs. Met treatment group; **(D)** Enriched bubble chart of KEGG pathway of differential genes in thyroid tissue, **(a)** different genes of mice in NC group vs. HT group, **(b)** different genes of mice in HT group vs. Met treatment group.
