# Supplementary material for: Metformin Reverses Hashimoto’s Thyroiditis by Regulating Key Immune Events
Source: Front Cell Dev Biol. 2021 May 28;9:685522. doi: 10.3389/fcell.2021.685522 (PMC8193849; doi:10.3389/fcell.2021.685522)
Supplement: Supplementary file 1 [file Table_1.DOCX]

**Table S1. Different species at each classification level.**

| **Classification** | **Bacterium** | **Mean (HT)** | **SD (HT)** | **Mean (MET)** | **SD (MET)** | **Mean (NC)** | **SD (NC)** | ***P* value** | **FDR** |
| --- | --- | --- | --- | --- | --- | --- | --- | --- | --- |
| Class | Coriobacteriia | 2.001 | 1.018 | 1.039 | 0.258 | 0.536 | 0.282 | 0.007 | 0.067 |
|  | Mollicutes | 0.310 | 0.335 | 0.283 | 0.125 | 0.000 | 0.000 | 0.016 | 0.067 |
|  | TM7_3 | 2.621 | 3.029 | 3.508 | 4.019 | 0.000 | 0.000 | 0.016 | 0.067 |
|  | Unclassified | 0.069 | 0.054 | 0.066 | 0.040 | 0.000 | 0.000 | 0.017 | 0.067 |
|  | Erysipelotrichi | 4.469 | 2.892 | 5.179 | 4.344 | 0.590 | 0.378 | 0.026 | 0.083 |
| Family | Coriobacteriaceae | 2.001 | 1.018 | 1.039 | 0.258 | 0.536 | 0.282 | 0.007 | 0.060 |
|  | Paraprevotellaceae | 1.011 | 0.507 | 2.183 | 1.312 | 0.001 | 0.001 | 0.011 | 0.060 |
|  | Bacteroidaceae | 0.614 | 0.419 | 1.284 | 0.798 | 0.005 | 0.006 | 0.011 | 0.060 |
|  | Prevotellaceae | 0.704 | 0.623 | 0.438 | 0.379 | 0.000 | 0.000 | 0.013 | 0.060 |
|  | F16 | 2.621 | 3.029 | 3.508 | 4.019 | 0.000 | 0.000 | 0.016 | 0.060 |
|  | Turicibacteraceae | 8.155 | 10.295 | 5.361 | 4.706 | 0.000 | 0.000 | 0.016 | 0.060 |
|  | Odoribacteraceae | 0.162 | 0.190 | 0.196 | 0.207 | 0.006 | 0.012 | 0.016 | 0.060 |
|  | Clostridiaceae | 0.170 | 0.178 | 0.147 | 0.187 | 0.002 | 0.003 | 0.017 | 0.060 |
|  | Peptostreptococcaceae | 0.403 | 0.268 | 0.572 | 0.770 | 0.001 | 0.001 | 0.018 | 0.060 |
|  | Erysipelotrichaceae | 4.469 | 2.892 | 5.179 | 4.344 | 0.590 | 0.378 | 0.026 | 0.078 |
|  | Christensenellaceae | 0.000 | 0.000 | 0.006 | 0.007 | 0.000 | 0.000 | 0.043 | 0.118 |
| Genus | Pediococcus | 0.197 | 0.124 | 0.114 | 0.086 | 0.000 | 0.000 | 0.010 | 0.105 |
|  | Bacteroides | 0.614 | 0.419 | 1.284 | 0.798 | 0.005 | 0.006 | 0.011 | 0.105 |
|  | Prevotella | 1.712 | 0.914 | 2.618 | 1.588 | 0.001 | 0.001 | 0.015 | 0.105 |
|  | Turicibacter | 8.155 | 10.295 | 5.361 | 4.706 | 0.000 | 0.000 | 0.016 | 0.105 |
|  | Odoribacter | 0.162 | 0.190 | 0.196 | 0.207 | 0.006 | 0.012 | 0.016 | 0.105 |
|  | Allobacμl um | 4.276 | 2.827 | 4.831 | 4.491 | 0.442 | 0.480 | 0.027 | 0.143 |
| Order | Coriobacteriales | 2.001 | 1.018 | 1.039 | 0.258 | 0.536 | 0.282 | 0.007 | 0.060 |
|  | RF39 | 0.270 | 0.339 | 0.249 | 0.132 | 0.000 | 0.000 | 0.015 | 0.060 |
|  | CW040 | 2.621 | 3.029 | 3.508 | 4.019 | 0.000 | 0.000 | 0.016 | 0.060 |
|  | Turicibacterales | 8.155 | 10.295 | 5.361 | 4.706 | 0.000 | 0.000 | 0.016 | 0.060 |
|  | Unclassified | 0.239 | 0.163 | 0.169 | 0.063 | 0.000 | 0.000 | 0.017 | 0.060 |
|  | Erysipelotrichales | 4.469 | 2.892 | 5.179 | 4.344 | 0.590 | 0.378 | 0.026 | 0.078 |
| Phylum | Unclassified | 0.039 | 0.021 | 0.029 | 0.008 | 0.000 | 0.000 | 0.015 | 0.054 |
|  | Tenericutes | 0.310 | 0.335 | 0.283 | 0.125 | 0.000 | 0.000 | 0.016 | 0.054 |
|  | TM7 | 2.621 | 3.029 | 3.508 | 4.019 | 0.000 | 0.000 | 0.016 | 0.054 |
| Species | Bacteroides_acidifaciens | 0.193 | 0.132 | 0.524 | 0.350 | 0.005 | 0.006 | 0.010 | 0.162 |
|  | Ruminococcus_flavefaciens | 0.027 | 0.036 | 0.029 | 0.039 | 0.000 | 0.000 | 0.034 | 0.258 |
|  | Butyricicoccus_pul licaecorum | 0.000 | 0.001 | 0.005 | 0.006 | 0.001 | 0.002 | 0.045 | 0.611 |

HT: Hashimoto’s thyroiditis group; MET: metformin treatment group; NC: normal control group; SD, standard deviation; FDR: false discovery rate.
