# Supplementary figures and images for: Metformin Reverses Hashimoto’s Thyroiditis by Regulating Key Immune Events
Source: Front Cell Dev Biol. 2021 May 28;9:685522. doi: 10.3389/fcell.2021.685522 (PMC8193849; doi:10.3389/fcell.2021.685522)

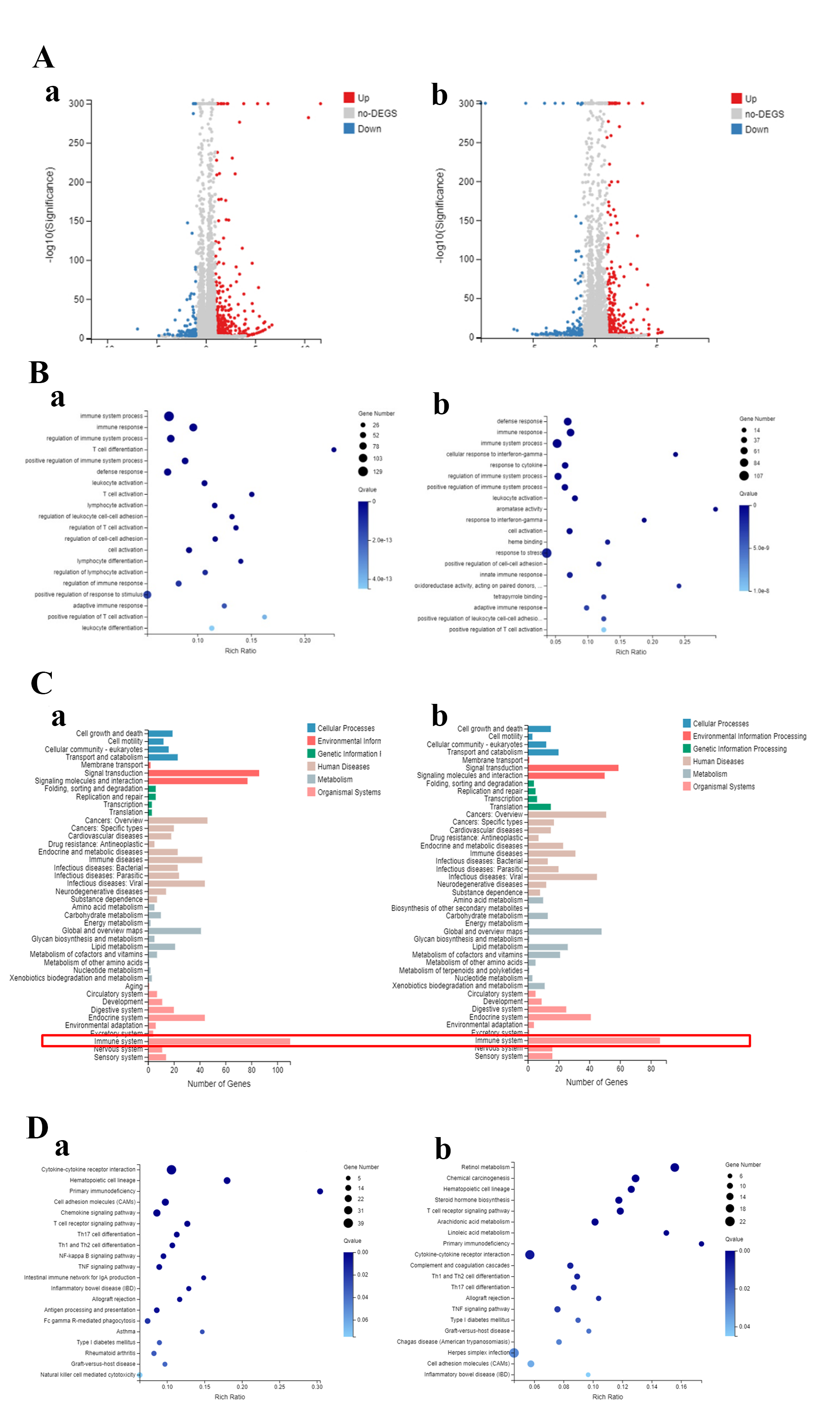

Supplement: Supplementary Figure 1 — mRNA sequencing of mice thyroid tissue. (A) Thyroid tissue differential gene volcano map. (a) different genes of mice in NC group vs. HT group, (b) different genes of mice in HT group vs. Met treatment group; (B) GO enrichment bubble chart of differential genes in thyroid tissue. (a) different genes of mice in NC group vs. HT group, (b) different genes of mice in HT group vs. Met treatment group; (C) KEGG pathway classification of differential genes in thyroid tissue. (a) different genes of mice in NC group vs. HT group, (b) different genes of mice in HT group vs. Met treatment group; (D) Enriched bubble chart of KEGG pathway of differential genes in thyroid tissue, (a) different genes of mice in NC group vs. HT group, (b) different genes of mice in HT group vs. Met treatment group. [file Image_1.TIF]
